# A plea for descriptive social ontology

**DOI:** 10.1007/s11229-023-04263-7

**Published:** 2023-08-18

**Authors:** Kathrin Koslicki, Olivier Massin

**Affiliations:** Faculté des Lettres et Sciences Humaines, Institut de Philosophie, University of Neuchâtel, Espace Tilo-Frey, 2000 Neuchâtel, Switzerland

**Keywords:** Descriptive metaphysics, Social ontology, Explanation, Description, Phenomenology, Revisionary metaphysics, Appearances, Metametaphysics

## Abstract

Social phenomena—quite like mental states in the philosophy of mind—are often regarded as potential troublemakers from the start, particularly if they are approached with certain explanatory commitments, such as naturalism or social individualism, already in place. In this paper, we argue that such explanatory constraints should be at least initially bracketed if we are to arrive at an adequate non-biased description of social phenomena. Legitimate explanatory projects, or so we maintain, such as those of making the social world fit within the natural world with the help of, e.g., collective intentionality, social individualism, and the like, should neither exclude nor influence the prior description of social phenomena. Just as we need a description of the mental that is not biased, for example, by (anti)physicalist constraints, we need a description of the social that is not biased, for example, by (anti)individualist or (anti)naturalist commitments. Descriptive social ontology, as we shall conceive of it, is not incompatible with the adoption of explanatory frameworks in social ontology; rather, the descriptive task, according to our conception, ought to be recognized as prior to the explanatory project in the order of inquiry. If social phenomena are, for example, to be reduced to nonsocial (e.g., psychological or physical) phenomena, we need first to understand clearly what the social candidates for the reduction in question are. While such descriptive or naïve approaches have been influential in general metaphysics (see Fine 2017), they have so far not been prominent in analytic social ontology (though things are different outside of analytic philosophy, see esp. Reinach (1913). In what follows, we shall outline the contours of a descriptive approach by arguing, first, that description and explanation need to be distinguished as two distinct ways of engaging with social phenomena. Secondly, we defend the claim that the descriptive project ought to be regarded as prior to the explanatory project in the order of inquiry. We begin, in Section 2, by considering two different ways of engaging with mental phenomena: a descriptive approach taken by descriptive psychology and an explanatory approach utilized in analytic philosophy of mind. We take these two ways of approaching the study of the mind to be analogous to the distinction we want to draw in social ontology between a descriptive and an explanatory approach to the study of social phenomena. We consider next, in Section 3, how our approach compares to neighboring perspectives that are familiar to us from general metaphysics and philosophy more broadly, such as Aristotle’s emphasis on “saving the appearances”, Strawson’s distinction between descriptive and revisionary metaphysics, as well as Fine’s contrast between naïve and foundational metaphysics. In Section 4, we apply the proposed descriptive/explanatory distinction to the domain of social ontology and argue that descriptive social ontology ought to take precedence in the order of inquiry over explanatory social ontology. Finally, in Section 5, we consider and respond to several objections to which our account might seem to be susceptible.

## Introduction

Social phenomena—quite like mental states in the philosophy of mind—are often regarded as potential troublemakers from the start, particularly if they are approached with certain explanatory commitments, such as naturalism or social individualism, already in place. In this paper, we argue that such explanatory constraints should be at least initially bracketed if we are to arrive at an adequate non-biased description of social phenomena. Legitimate explanatory projects, or so we maintain, such as those of making the social world fit within the natural world with the help of, e.g., collective intentionality, social individualism, and the like, should neither exclude nor influence the prior description of social phenomena. Just as we need a description of the mental that is not biased, for example, by (anti)physicalist constraints, we need a description of the social that is not biased, for example, by (anti)individualist or (anti)naturalist commitments.

Descriptive social ontology, as we shall conceive of it, is not incompatible with the adoption of explanatory frameworks in social ontology; rather, the descriptive task, according to our conception, ought to be recognized as prior to the explanatory project in the order of inquiry. If social phenomena are, for example, to be reduced to non-social (e.g., psychological or physical) phenomena, we need first to understand clearly what the social candidates for the reduction in question are. While such descriptive or naïve approaches have been influential in general metaphysics (see Fine, 2017 for a recent reassessment), they have so far not been prominent in analytic social ontology (though things are different outside of analytic philosophy, see esp. Reinach, 1913). In what follows, we shall outline the contours of a descriptive approach by arguing, first, that description and explanation need to be distinguished as two distinct ways of engaging with social phenomena. Secondly, we defend the claim that the descriptive project ought to be regarded as prior to the explanatory project in the order of inquiry.

We begin, in Sect. 2, by considering two different ways of engaging with mental phenomena: a descriptive approach taken by descriptive psychology and an explanatory approach utilized in analytic philosophy of mind. We take these two ways of approaching the study of the mind to be analogous to the distinction we want to draw in social ontology between a descriptive and an explanatory approach to the study of social phenomena. We consider next, in Sect. 3, how our approach compares to neighboring perspectives that are familiar to us from general metaphysics and philosophy more broadly, such as Aristotle’s emphasis on “saving the appearances”, Strawson’s distinction between descriptive and revisionary metaphysics, as well as Fine’s contrast between naïve and foundational metaphysics. In Sect. 4, we apply the proposed descriptive/explanatory distinction to the domain of social ontology and argue that descriptive social ontology ought to take precedence in the order of inquiry over explanatory social ontology. Finally, in Sect. 5, we consider and respond to several objections to which our account might seem to be susceptible.

## An analogy: descriptive vs. explanatory philosophy of mind

### Two traditions in the philosophy of mind

Two chief philosophical traditions have investigated the mind in the 20th century. Analytic philosophers have studied the mind under the label “philosophy of mind”; philosophers in the tradition of Brentano have approached mental phenomena under the label “descriptive psychology” (also referred to as “phenomenology”). The most striking contrast between analytic philosophy of mind and descriptive psychology pertains to their relation to the mind/body problem, viz., the question of how to understand the relationship between mental episodes and their physical correlates.

Analytic philosophy of mind has been largely centered around the mind/body problem. Interest in mental phenomena has typically been driven by their implications for how the mind and the body are related to one another. Thus, pain, emotions, color perception, consciousness, or phenomenal character have typically received attention because they constitute potential troublemakers for physicalism. By contrast, descriptive psychologists, including Brentano, Meinong, Stumpf, Reinach, Husserl, and Scheler, among others, proposed an impressive number of claims and theories about the mind while remaining nearly silent about the mind-body problem, and deliberately so. Brentano, and many others following him, thought that descriptive psychology should avoid reference to physiological processes:

[Descriptive psychology] will therefore, even in its highest state of perfection, never mention a physico-chemical process in any of its doctrines. (Brentano, 2002, p. 4).

The first difference between analytic philosophy of mind and descriptive metaphysics is thus a difference of interest. Descriptive psychologists want to arrive at a precise mapping of the different psychological phenomena as they appear to us, e.g., their relations, their differences, and their commonalities, and are interested in the relations between psychological phenomena and their physical correlates only insofar as understanding this relation helps to complete such a map. Analytic philosophers of mind, for their part, want most to understand the relation between the mind and the body, and are interested in mental phenomena primarily within this context. It is sometimes suggested that this contrast is at bottom a divergence between anti-naturalist and naturalist approaches to the mind: descriptive psychology, according to this characterization, would be fundamentally anti-naturalist, while philosophy of mind would tend towards naturalism. While it is true that descriptive psychologists are largely anti-naturalists and that a majority of philosophers of mind are naturalists,^1^ this proposal poorly captures the real distinction between the two approaches. A more plausible way to capture this distinction, in our view, is to maintain that the two approaches are involved in different projects. Descriptive psychology, on the one hand, aims mostly to *describe* mental phenomena as they appear to us; analytic philosophy of mind, on the other hand, is concerned primarily with *explaining* mental phenomena in causal or metaphysical terms, even if the resulting explanations diverge from what is immediately apparent to us. There are, clearly, exceptions on both sides, but we maintain that this broad characterization nevertheless captures dominant strands in these two traditions. Our interest is not here in the history of these philosophical schools, but in demarcating two different kinds of philosophical projects that are relevant to the philosophical study of the mind and, ultimately, as we will argue below, to the study of social phenomena.

### Descriptive vs. explanatory projects

Our first claim is that philosophy of mind subsumes two different projects under it: descriptive philosophy of mind and explanatory philosophy of mind. Descriptive philosophy of mind aims at capturing mental phenomena as they appear to us, by making explicit the various distinctions, relations, and commonalities they exhibit. Descriptive philosophy of mind consists of claims of the form, “It appears to be the case that p” or “p, as it appears” (see below for a justification of this appositional formulation), where p is a proposition about some mental phenomena, such as the following: “It appears to be the case that desires and beliefs are distinct”; “All perceptions are intentional, as it appears”; “One cannot remember something that did not happen, as it appears”; “Fearing × depends on being presented with ×, as it appears”; and the like.

Explanatory philosophy of mind, on the other hand, starts from claims about mental appearances and submits them to critical scrutiny. There are two main ways of doing so. Explanatory philosophers of mind may, first, take the appearances described by descriptive psychology to be veridical, and set out to explain their content; alternatively, they may, as a second strategy, take appearances to be illusory, and subsequently attempt to explain why we have such illusory appearances. To illustrate, consider the descriptive claim, “Phenomenal consciousness exists, as it appears”. Disagreements about the truth of this sentence are part of descriptive psychology. Consider now philosophers of mind who are engaged in an explanatory project and who agree that the descriptive claim is true. Some among them will consider the appearances reported in the descriptive sentence to be veridical. Thus, they will accept that phenomenal consciousness exists and that this phenomenon constitutes (part of) their explanandum. They will, typically, try to elucidate how phenomenal consciousness is related to brain states. But other explanatory philosophers who also agree with the descriptive claim will instead discard the appearance in question as illusory. For them, consciousness does not in fact exist and, therefore, does not call for an explanation. Illusionism about consciousness, however, still needs to explain the illusory appearance that consciousness exists: this erroneous impression, for the illusionist, is (part of) what needs to be explained.

Let us call explanatory philosophers of mind who take mental appearances to be veridical *foundationalists*, and those who take mental appearances to be deceptive *revisionists*. Identity-theories, functionalism, dualism, Russellian monism, to mention but a few, are foundationalist theories. Eliminativism about mental states (Churchland, 1981), illusionism about consciousness (Dennett, 1991; Frankish, 2016; Kammerer, 2021), or eliminativism about pain (Hardcastle, 2001) are revisionary theories either of the mind in its entirety or of some part or aspect of it. Both foundationalist and revisionary approaches are in the business of saving the appearances captured by descriptive metaphysics. But while foundationalists philosophers of mind want to save the content of the appearances, revisionary philosophers of mind consider such contents to be unsalvageable: only the appearing of such contents can be saved.


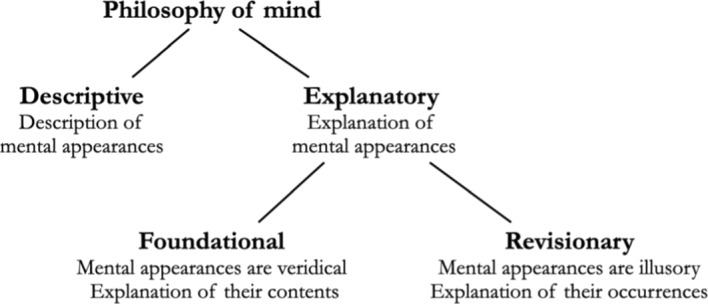


In practice, claims do not wear their descriptive or revisionary status on their sleeves. Consider the view that perception is not intentional in the sense that there is no distinction between perceptual acts and perceptual objects. This view may be defended as a revisionary view: perception seems to be intentional, but is in fact not (Dennett, 1987). Alternatively, it can also be intended as a descriptive thesis: perception does not seem to be intentional (as is argued, for example, by neutral monists such as Russell, 1919, 1995: pp. 142–143; James, 1912 or Carnap, 1928); the act/object distinction, according to this conception, is not part of the appearances. On the former revisionary view, the intentionalist description of mental appearances is correct, but the appearances in question are illusory. According to the latter descriptive view, Brentano and his followers misdescribed mental appearances. Importantly, heterodox descriptive claims should not be conflated with revisionary claims: disagreement about the right description of appearances is distinct from disagreement about the veridicality of appearances.

### The priority of description

Both the descriptive and the explanatory projects are legitimate and important. How are they related? Our second claim is that the descriptive project is prior in the order of inquiry to the explanatory project. Three cognate arguments support this priority. The first is reminiscent of Meno’s paradox (otherwise known as the paradox of inquiry). If we are to explain the mental, we must at least have a sufficient grasp of the mind in order to be able to know what to look for. At the same time, there must also be other aspects of the mind of which we are ignorant; otherwise, no explanation would be needed. The answer is given by descriptive philosophy of mind: prior to the explanatory project, we know how the mental appears to us; but we lack knowledge as to whether these appearances are veridical and what explains them (be it their content or their occurrence). The second argument pertains to the evaluation of explanations. Not only is the descriptive task crucial for fixing the explanandum from the start, it is also needed to assess the success or failure of the proposed explanation. Such an evaluation is impossible without an independent grasp of what it is that we are trying to explain (e.g., reduce, identify, ground, eliminate, etc.). The third argument pertains to the identification of our subject-matter. Absent a clear identification of the subject of the inquiry, one can neither know whether an explanation has resulted in a change of the subject-matter nor whether distinct explanations target the same explanandum. For this reason, it is vital to ensure that we first correctly identify the mental; and, secondly, that we do not overlook important features of it.

Descriptive psychology does just this: it characterizes the mental realm, and thereby provides explanatory philosophers of mind with their explananda. This is true for both versions of explanatory philosophy of mind. Within the foundationalist camp, reductionist philosophers of mind will view descriptions as yielding candidates for reduction, while dualists will view these candidates as not suitable for reduction. Revisionary philosophy of mind is no less dependent on descriptive psychology. For if we are to classify some appearances as illusory, we first need to be clear on what these appearances are. We need to agree, for example, that the Müller-Lyer lines look to be of different lengths (a descriptive claim) before we then go on to diagnose this appearance as defective. Likewise for mental phenomena.

### Two fallacies

Failing to distinguish sharply between descriptive and explanatory philosophy of mind can lead to two important fallacies. The first was spotted by early identity theorists and dubbed “the phenomenological fallacy” by Place (1956). The phenomenological fallacy, broadly construed, consists in going beyond appearances by drawing ontological conclusions directly from the description of appearances. The theory of sense data, which claims that immediately perceived sensory objects are mind-dependent entities, is often considered to be an instance of this fallacy. From the fact that we seem to see a green object, while there is none in front of us, we cannot conclude, without further argument, that there is a green object before our mind, viz., a sense datum. Another instance of the phenomenological fallacy is when descriptive psychologists claim that the description of mental episodes targets the essences of such episodes. While such a claim may be true, it is not a descriptive but an explanatory claim. At best, essentialist descriptive claims might be of the form, e.g., “It appears to be part of the essence of emotions that they are either positive or negative”. But whether this characteristic is indeed part of the essence of emotions is a question that cannot be decided on the basis of the appearances alone. Description, by itself, does not settle the explanatory challenges at issue.

There is a reverse fallacy, which we call the “explanatory fallacy”. (Fine, 2017 uses the term, “foundationalist fallacy”, to refer to the same phenomenon.) This is the fallacy of letting explanatory concerns shape the description of the phenomena under investigation. A good description should be immune from any inclination as to how the explanatory challenges at issue are ultimately to be settled. The *explanans* should fit the *explanandum*, not the reverse. Our description of what is to be explained should not be biased by the candidate explanation we may favor, on pain of ending up like the person who is looking for just what she found, whatever it is. For instance, if the reason why we describe desires in terms of their functional role is that we think that functionalism is the correct response to the mind-body problem, we are guilty of committing the explanatory fallacy. Early identity theorists who correctly drew attention to the phenomenological fallacy may themselves have fallen prey to the explanatory fallacy. Smart (1959, 2007) insisted that the descriptions of our sensations should be *topic-neutral*, in the sense of being committed neither to physicalism nor to its negation. But from a descriptive standpoint, such a requirement is an unwarranted interference of explanatory constraints with the descriptive project. For why should the description of what is to be explained leave all explanations open? The explanans should fit the description, not the reverse. Descriptions should be neutral indeed, but in the sense of not being influenced by any explanatory commitments.

The descriptive psychologist, upon encountering the results of research in the philosophy of mind, might complain that the few descriptions he finds there are nearly all botched and biased. The philosopher of mind, in turn, will likely be annoyed by the way descriptive psychologists beat around the bush by drawing countless seemingly scholastic distinctions while failing to answer the explanatory question of how mental phenomena are related to physical phenomena. The truth is that proponents of these two approaches are involved in distinct and complementary projects. But while descriptive psychology without philosophy of mind is merely incomplete, philosophy of mind without descriptive psychology is simply impossible. Since we cannot engage in the explanatory project without first having fixed our explanandum, the best strategy is to confront this task explicitly and thoroughly.

## Descriptive vs. explanatory metaphysics

### Previous versions of the distinction

Although descriptive or naïve approaches so far have not taken hold in analytic social ontology, such approaches have been influential in general metaphysics and in philosophy more broadly. Arguably, Aristotle, when approaching a new subject-matter, tends to favor a descriptive approach which allows him, even in his considered views, to “save the appearances” as much as possible. In *Nicomachean Ethics* VII.2, for example, at the beginning of his discussion of weakness of the will (*“akrasia”*), Aristotle describes his method as follows:

As in the other cases we must set out the appearances [*phainomena*], and first of all go through the puzzles. In this way we must prove the common beliefs about these ways of being affected – ideally, all the common beliefs, but if not all, most of them, and the most important. For if the objections are solved, and the common beliefs are left, it will be an adequate proof. [NE VII.2, 1145b2-7, transl. by Terence Irwin].

When Aristotle reports what appears (*“phainesthai”*) or seems (*“dokein”*) to be the case, the appearances in question, which may result from either perception or reason, also include commonly accepted beliefs (“*endoxa”*) (Irwin, 1999, p. 317). At least initially, Aristotle neither endorses nor rejects the appearances he describes, but rather uses them as starting-points for his arguments, as the passage just cited indicates. The goal is to save the appearances, as much as possible; and, to the extent that not all of the appearances can be preserved, those that are deemed to be the most important ones among the appearances initially set out should be retained.

Although descriptive approaches are less common in analytic philosophy, at least three analytic schools of thought emphasize the value of description. First, ordinary language philosophers, under the influence of Wittgenstein, developed detailed descriptions of linguistic practices (see Cappelen and McKeever, 2022, for a recent re-evaluation). Secondly, Strawson (1959) famously distinguished between “descriptive” and “revisionary” metaphysics and seems to prefer the former to the latter. According to Strawson, “[d]escriptive metaphysics is content to describe the actual content of our thought about the world, revisionary metaphysics is concerned to produce a better structure” (Strawson, 1959, p. 10). While ordinary language philosophers see descriptions as targeting ordinary linguistic practices, Strawson views descriptions as bearing on our conceptual scheme. A third kind of descriptive approach takes itself to be directed towards *essences*, rather than aiming to provide descriptions of our *language* or *concepts*. (A similar distinction between descriptive projects is discussed in Hacker, 2009, p. 137).

While the project of describing essences was highly popular in the aftermath of Brentano, such an essentialist descriptive approach is less widespread within contemporary analytic philosophy. This may be due first to skepticism towards essences and, second, to a relative lack of popularity of descriptive projects. In recent times, however, Fine (2017) has argued for a distinction between naïve and foundational metaphysics and similarly assigns priority, in some respects, to naïve metaphysics over foundational metaphysics. Both naïve and foundational metaphysics, in Fine’s view, share a common interest in the nature or essence of things; but naïve metaphysics proceeds by studying the appearances, i.e., the world as it presents itself to us, while foundational metaphysics aims to discern the reality that lies behind these appearances. Naïve metaphysics, for Fine, can be pursued more or less independently of foundational metaphysics: thus, in investigating questions concerning the nature of numbers or sets, for example, one need not settle questions concerning their reality. When Fine speaks of “reality”, here, he does not have in mind “existence”; rather, when something is really the case, in the sense at issue, the phenomenon in question is part of the “ultimate furniture of the world”, i.e., truths concerning the types of entities in question must be included in a complete description of the world. In fact, in cases of conflict, Fine advises that the claims of naïve metaphysics, if anything, should be given precedence over those of foundational metaphysics. There is in general a danger, so Fine warns, that premature attempts to address foundational questions will lead to a distorted depiction of reality if the subject-matter in question is not first investigated properly from a naïve point of view. Both realists and anti-realists concerning a given phenomenon might agree on the need to “save the appearances”; but what this comes to can only be appreciated once we have determined, from the point of view of naïve metaphysics, what the appearances are that are supposedly worth saving.

The distinction between naïve and foundational metaphysics Fine is after is not one between the “ordinary” (or “pre-philosophical”) and the “philosophical”, since for Fine no clear dividing line can be drawn between what is part of philosophy and what is part of our everyday life. Unlike Strawson’s distinction between descriptive and revisionary metaphysics, Fine’s distinction between naïve and foundational metaphysics is not primarily aimed at characterizing our conceptual scheme or the structure of our thought. In contrast to Strawson, Fine does not view naïve and foundational metaphysics as competitors; rather, they are meant to complement each other, since both are directed at studying the same world, but through different lenses, one by focusing on the appearances and the other by aiming to capture the reality that lies behind these appearances. Just as naïve metaphysics does not necessarily proceed by aligning itself with our ordinary language or thought, so similarly there is nothing built into foundational metaphysics as such that would privilege science over common-sense.

### Descriptive metaphysics

We agree with Strawson and Fine on the centrality of the meta-theoretical distinction between descriptive and non-descriptive metaphysics. Like them, we also believe that some priority must be given to the descriptive project. The way we propose to draw the distinction, however, significantly differs from each of their proposals.

Descriptive metaphysics, we contend, purports to describe the contents of appearances. We construe appearances very broadly as including perceptual appearances, inner and outer consciousness, memory, intuitions, affective presentations (e.g., something’s feeling dangerous), beliefs, opinions, expectations, etc. (Though beliefs are excluded by Huemer (2007), the resulting view is nevertheless closely related to ours.) Moreover, we consider appearances as being intentional: the appearing (the act) is distinct from what appears (the content). Finally, we assume that the content of an appearance is propositional (although not necessarily conceptual). As a consequence, appearance reports always involve an appearance operator of some kind: e.g., “it seems that p”; “it appears to be the case that p”; or “p appears to be the case”. Thus, appearances include whatever seems to be the case: that orange is more like red than like blue; that the Earth’s population is increasing; that some vegetables require more water to grow than others; that 2 + 3 = 5; that there are trees; and so forth.

Descriptive metaphysics describes what appears and not primarily the appearing of these phenomena, except when the appearing is itself taken to be part of the appearances in question. To stress this point, and building on Dancy (2000, p. 128ff), we prefer to use the following appositional construction of the appearance operator to capture the objects of descriptive metaphysics: “p, as it appears”; “p, by the look of things”; “p, seemingly”. It is indeed the contents of such statements that descriptive metaphysics seeks to describe properly, rather than the fact that they appear or their appearing. When p concerns mental states, the claim in question belongs to descriptive psychology. When p concerns numbers, the claim belongs to descriptive arithmetic. When p concerns social phenomena, the claim belongs to descriptive social ontology or descriptive social science. (Below, we take up the question of how to distinguish descriptive claims that belong to social ontology from empirical descriptive claims that are part of the social sciences.)

We saw that Strawson, Fine and ordinary language philosophers disagree about the target of descriptive metaphysics. Does descriptive metaphysics concern the structure of our language, our conceptual schemes, or essences? Insofar as these claims are construed as meta-philosophical claims about the nature of descriptive metaphysics, we take these three approaches to be equally mistaken. Descriptive metaphysics, properly construed, leaves such questions open. Whether appearances reflect essences, linguistic structures, a priori categories of our cognitive system, or yet other entities are questions that cannot be settled by descriptive metaphysics but only by explanatory metaphysics. As far as descriptive metaphysics is concerned, all we have are claims to the effect that, e.g., material objects appear spatially located; processes appear to have temporal parts; some properties (e.g., being red, being elegant, or being painful) appear to be gradable, while others (e.g., being obligatory, being president or being an odd number) appear not to be gradable. Such appearances can then be explained in many different ways. A realist will think that objects appear to be spatially located, because they are in fact spatially located, and that our cognitive system represents their location as it is. A Kantian will think that objects appear to be spatially located, because space is an a priori form of sensibility. Neither appearances nor their description can by themselves decide whether appearances are in fact about, e.g., worldly essences, a priori forms of sensibility, conceptual schemes, or linguistic projections.

This is not to say that descriptive metaphysics cannot contain claims about the existence or essence of some phenomena. But such claims are always under the scope of an appearance operator of some form, such as “x is essentially F, as it appears” or “x exists independently of its being perceived, as it appears”. In the latter case, the apparent lack of a dependence relation between the existence of a certain phenomenon and its being perceived might itself be presented to us as part of the appearance. As before, however, whether it really is the case that the phenomenon in question can exist independently of its being perceived can only be determined once the investigation has proceeded from the descriptive to the explanatory phase.

*Counterexamples* are one familiar kind of descriptive claim. A counterexample does not purport to explain appearances but aims to report appearances that allegedly clash with explanatory commitments. Once it is seen that counterexamples are descriptive claims, it becomes easier to spell out the variety of possible reactions one might have to counterexamples. Consider, for instance, the view that no two entities of the same kind can be at exactly the same place at the very same time. Sounds have been advanced as a counterexample to this view (Casati & Dokic, 1994): as it appears, two sounds can be at the same place at the same time, as is illustrated for example by a chord played on a guitar. Upholders of the initial view may reject the counterexample in three main ways. First, they might deny the existence of the appearance in question: it is not the case, they might argue, that sounds appear to be exactly in the same place at the same time, because sounds do not appear to have an exact location. Secondly, they might grant that there is such an appearance but argue that the appearance is deceptive, in which case it would still have to be explained why we are subject to the illusion in question. Thus, given this response, though sounds appear to be exactly co-located, sounds in fact occupy distinct locations, but the determination of their exact locations exceeds our sensory thresholds. Thirdly, one might grant that there is such an appearance and that it is veridical, but argue that the counterexample has misdescribed the appearance, so that, once properly described, the appearance no longer clashes with the explanatory framework in question. According to this third line of response, the correct way to describe co-located sounds is to maintain that sounds do not appear to *be* at the same place at the same time, but rather appear to *occur* at the same place simultaneously, which no longer contradicts the initial view (Hacker, 1982). Finally, upholders of the view under attack might grant the counterexample: that is, they might agree that there is such an appearance; that the appearance is veridical; and that it has been properly described in a way that contradicts their view. Proponents of this last response might instead opt to restrict their view: for example, they might want to allow that, while no two material objects can be in the same place at the same time, sounds do not belong to the category of material objects and are therefore not affected by the apparent counterexample in question.

### Explanatory metaphysics

We contrast descriptive metaphysics with explanatory metaphysics. Explanatory metaphysics, as we understand it, encompasses Finean foundational metaphysics as well as Strawsonian revisionary metaphysics. Explanatory metaphysics, as we conceive of it, is concerned not with how things appear, but with how things are. Explanatory metaphysics thus divides into two main branches: *foundational* metaphysics and *revisionary* metaphysics.

According to our usage, a metaphysician adopts a *foundational* stance with respect to some appearances when she takes the content of these appearances at face value and purports either to explain these contents or to provide a picture of the world which can accommodate these appearances. We use “explanation” here broadly to encompass a variety of different relations that might be said to hold among the phenomena in question or their descriptions, including reduction, identity, grounding, supervenience, functional realization, analysis, paraphrase, and even the position that a given content should be left unexplained, i.e., that things really just are as they appear to be, and that nothing needs to be added to, or taken away from, the description (as is illustrated, for example, by Campbell, 1993’s simple view of colors). This position, which consists in just endorsing the appearances as they are, and stopping there, is not a descriptive position, but an explanatory one. For the facts, if indeed they are facts, that the appearances are not deceptive and that nothing explains them are not presented in the appearances themselves.

We will say that a metaphysician adopts a *revisionary* stance with respect to some appearances, by contrast, when she takes the appearances in question to be deceptive. Consequently, while a metaphysician who chooses such a revisionary perspective does not need to explain the contents of the appearances in question, she must nevertheless explain why things appear to us differently from how they in fact are. For example, a Humean about personal identity, who denies that anything genuinely persists through time in the face of qualitative change, may invoke the human customary propensity to posit persisting persons when in fact there are only distinct bundles of perception as an explanation of why it nevertheless appears to us to be the case that one and the same person can persist through qualitative change.

Note that, though some explanatory metaphysicians may be more inclined towards revising appearances while others are more invested in retaining appearances, these characterizations, in their current formulation, are too general: it is possible to maintain that some appearances are veridical while others call for revisions. In practice, most explanatory frameworks in metaphysics will simultaneously advance both foundational and revisionary claims. This is in part because appearances may contradict each other, so that giving up some appearances, as is done by the revisionary metaphysician, is in practice often unavoidable.


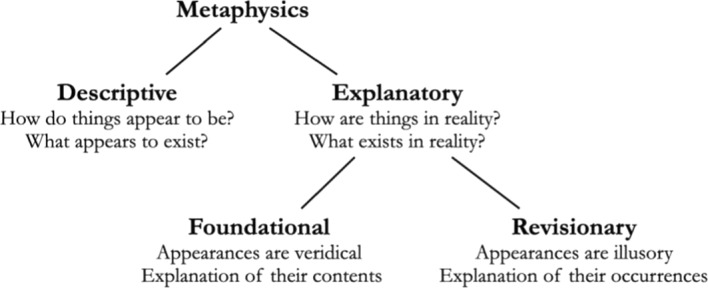


As in the philosophy of mind, it is important to draw a distinction within metaphysics as well between revisionary claims and heterodox descriptive claims. The revisionary or foundational status of a claim is not intrinsic to it, but depends on the descriptive position that is adopted by its proponent. Consider, for example, the claim that matter does not exist. Most philosophers would consider this to be a revisionary claim. Berkeley, however, maintains that his assertion that matter does not exist is not revisionary but foundational. In his view, the world present itself to us in such a way as to suggest that matter does not exist; relatedly, so Berkeley holds, the thesis that matter does not exist is “most agreeable to commonsense” (Berkeley, 1843, p. 172). Thus, the claim that matter does not exist, on this conception, follows from these two commitments: first, the descriptive claim that, as it appears, matter does not exist; and, secondly, the explanatory claim that appearances should be taken at face value. Berkeley’s descriptive claim, that matter appears not to exist, is not a revisionary claim, but an unorthodox descriptive claim. As a result, Berkeley’s explanatory thesis, that matter does not exist, is not revisionary, but foundational. By contrast, the claim that matter exists, in Berkeley’s view, should be regarded as revisionary, rather than foundational.

### The priority of descriptive metaphysics

Descriptive metaphysics is prior in the order of explanation to both foundational and revisionary metaphysics. Descriptive metaphysics is prior to foundational metaphysics: for in order to be able to assess the success of an explanation, one must first be clear about what it purports to explain. To accomplish this goal, an accurate description of the explanandum is needed. Descriptive metaphysics is also prior to revisionary metaphysics: for if we are going to fix defects in our existing ways of representing the world, we first need to be clear on how the world presents itself to us.

Descriptive metaphysics, in our view, is prior to explanatory metaphysics not in a normative but in a factual sense. This factual priority is not the same as the normative priority often advocated by commonsense philosophers. On this latter view, descriptive claims that reflect commonsense beliefs are not only prior in the order of explanation but also enjoy a higher epistemic status. On a strong version of this epistemic priority, commonsense beliefs are held not to be revisable on philosophical grounds (Boulter, 2007); on a weaker version, commonsense beliefs only have default justification and shift the burden of proof to their opponents (Lycan, 2019; Guillon, 2021). Our priority claim is even weaker than this latter view. It is one thing to claim that we cannot avoid starting with a description of the appearances; it is quite another thing to claim that the descriptive enjoys some sort of normative priority, even if only in a weak sense. From the fact that appearances are our inescapable starting point, it does not follow that these appearances are accurate or trustworthy. Our plea for descriptive metaphysics in general, and descriptive social ontology in particular, does not hang on a positive prejudice towards commonsense, although it is compatible with it. As a result, given our proposal, the dispute in question between broadly Moorean philosophers, who hold that appearances enjoy some presumption of truth, and revisionary philosophers, who believe that there is no good reason to ascribe normative priority to appearances (see, e.g., Cappelen, 2020), will have an impact on explanatory metaphysics, but does not affect descriptive metaphysics.

The descriptive and the explanatory projects thus differ with respect to their goals: the former aims at providing *descriptions*, while the latter attempts to produce *explanations*. Given this difference with respect to their aims, we also expect these two approaches to be guided by distinct criteria of success. Thus, we might, on the one hand, judge a description to be good if it is *adequate* and *comprehensive*, i.e., if it fits the appearances and does not leave out important distinctions and relations that are found in the phenomena at hand. An explanatory approach, on the other hand, might invoke such criteria as *parsimony* and *powerfulness*, in the sense that a good explanation should explain as much as possible with the fewest explanatory posits possible. By contrast, we may not be particularly concerned with parsimony while being engaged in the project of providing adequate and exhaustive descriptions of the phenomena under investigation. Given these distinct goals and criteria for evaluating success, it is furthermore natural to take the activities of describing, on the one hand, and explaining, on the other hand, to require different sets of skills from agents who engage in them. Providing good descriptions, on the one hand, might require an agent to exhibit *acuity*, i.e., keenness of observation and the ability to recognize distinctions which might otherwise be easily missed. An agent who is skilled at providing good explanations, on the other hand, might be characterized by *ingenuity*, e.g., the ability to combine the fewest explanatory elements possible to yield the largest possible explanatory gains.

## Descriptive vs. explanatory social ontology

### Descriptive social ontology

The very same distinctions, we maintain, apply within social ontology: 
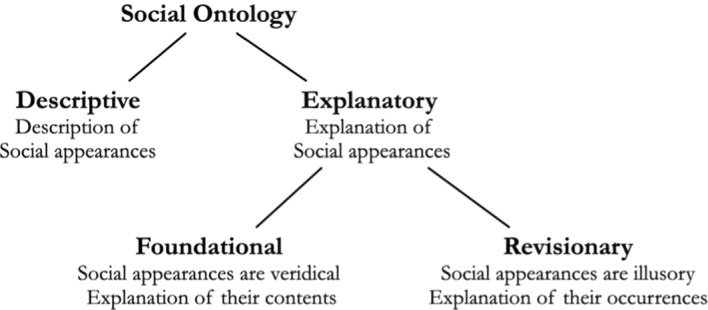


In social ontology, as in philosophy of mind as well as in general metaphysics, one should carefully distinguish the task of describing social appearances, from the task of explaining them, by adopting a foundational or a revisionary approach. Here again, we maintain, that the description of social phenomena is prior in the order of inquiry to their explanation. Things are more complicated in the case of social ontology, however, for two reasons.

First, social phenomena are not pre-theoretically well-delineated. What counts as social or not will depend on the general theory the social one endorses. Given this, the very extension of the field of social ontology seems to depend on prior theoretical commitments about the nature of social phenomena. As a result, descriptive social ontology appears to presuppose a general account of the social in order to determine which phenomena should be described in the first place. If true, the idea of describing appearances prior to endorsing certain explanatory commitments would then be doomed to fail: any description of the social seems to rely on a prior explanation of the nature of the social realm, in order to delineate which phenomena fall within the scope of descriptive social ontology.^2^

Our answer to this worry is to argue that, as far as a descriptive social ontology is concerned, a “bottom-up” approach to social phenomena is preferable to a “top-down” approach. Top-down approaches start by asking what all and only social phenomena have in common in virtue of which they are social. Many existing accounts of social reality take this line and aim at unifying social phenomena under a common explanatory scheme (Epstein, 2016; Guala & Hindriks, 2015; Lewis, 1969; Searle, 1995, 2010, to mention but a few). In contrast to these top-down approaches, descriptive social ontology, in our view, is better served by embracing a bottom-up strategy. Instead of beginning with an examination of the entire *social genus* as a whole, bottom-up descriptive social ontology proceeds by focusing on a broad range of specific types of social phenomena. Not much hangs on the sense in which these phenomena are social at the outset: “social phenomena”, according to this usage, can just be taken to mean phenomena that are regarded as “social” in some admittedly loose pre-theoretical sense of the term. Vague as this sense may be, it is sufficient to exclude, e.g., the study of molecules, black holes, or the auditory system from social ontology. One chief interest of a bottom-up approach for descriptive social ontology is to leave as open as possible further explanatory moves about the unity of social phenomena. In particular, a bottom-up approach initially leaves open the possibility that what unifies social phenomena is neither some one property that is shared by all and only social phenomena nor a relation that all and only social phenomena bear to some common source (e.g., collective intentionality). Rather, the social realm may simply turn out to be characterized by a plurality of essential distinctions and connections. If this latter possibility were to obtain, social phenomena would form a “flat” network consisting of various essential interconnections, no single one of which bears the responsibility of unifying the whole lot. If social reality were to fit this mold, then it would seem that only a bottom-up approach to the description of social phenomena would be able to uncover that this is in fact the structure of the social world.

The second reason that complicates the application of the descriptive/explanatory distinction to the field of social ontology stems from the different uses of the term, “descriptive”, within this discipline.^3^ Indeed, “descriptive” has recently come to be used in a distinct way in social ontology. In the aftermath of Haslanger (2000, 2005, 2006)’s influential work, descriptive social ontology is often contrasted, not with *explanatory* social ontology, but with *ameliorative* social ontology. How does ameliorative social ontology fit into our mapping of this discipline? We propose that ameliorative social ontology should not be viewed as a branch of explanatory social ontology. Explanatory social ontology is a theoretical endeavor, in the sense that it aims at epistemic values, such as truth, knowledge, or understanding. By contrast, the goal of ameliorative social ontology is not truth or other such epistemic values, but rather non-epistemic values, such as moral goodness, justice, or freedom. Ameliorative social ontology aims at providing theories and concepts that are helpful for the purpose of bringing about social change (or preserving social stability). Thus, the aim of explanatory social ontology is to discover truths about the social world, whereas the aim of ameliorative social ontology is to change or preserve (aspects of) the social world. Based on Aristotle’s distinction between the theoretical and practical sciences (*Metaphysics*, 1025b25, 1026a18–19, 1064a16–19, b1–3), we propose to distinguish descriptive and explanatory social ontology, on the one hand, as branches of theoretical social ontology, from ameliorative social ontology and its cognates, on the other hand, as branches of practical social ontology: 
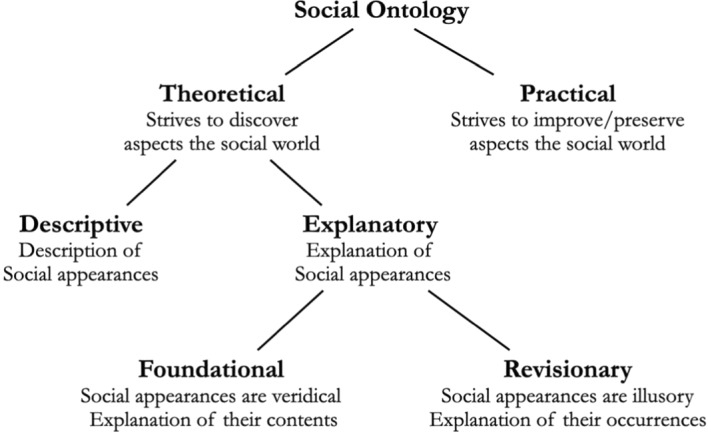


While the distinction between theoretical and practical social ontology has been one important focus of attention in recent years, as we just saw, the distinction between descriptive and explanatory social ontology has not so far received much systematic attention. It can, however, be spotted in various places in the literature, among them the following.

First, as in the philosophy of mind and in general metaphysics, philosophers belonging to the Brentanian tradition are more descriptively inclined in their approach to social phenomena than those belonging to competing traditions. Reinach (1989a [1913])’s account of speech acts (e.g., promising, ordering, granting, or submitting), of ownership and property rights, or of legal representation constitutes a remarkable achievement in this area. Scheler (1916)’s descriptive account of the varieties of social unities (e.g., herds, communities, societies, or collective persons) is another instance of a prolific descriptive social ontology, as is Stein (1925)’s descriptive account of the State, of its sovereignty, of its actions, and of the way it enacts laws (see Taieb, 2020, for recent discussion). Reinach, Scheler and Stein are not just descriptive social ontologists: all three assume, furthermore, that essentialist claims about, e.g., speech acts, social unities or the State, can be derived from a description of social appearances. This, on our account, is an explanatory claim that goes beyond the mere description of social appearances. Had these phenomenologists moved on without argument from the description of such intuitive claims about essences to essentialist conclusions, they would have been guilty of the phenomenological fallacy. This, however, is not how these theorists proceed: all of them propose arguments to the effect that appearances concerning essences should be taken seriously (see in particular Reinach, 1989b).

By contrast, few philosophers in the analytic tradition have adopted a mainly descriptive approach of social phenomena. Among the most notable exceptions to this generalization, though, are Austin (1975)’s description of the structure of what Reid (2011 [1788], chapter VI) called social acts^4^ and Hart’s descriptive approach to legal theory (see in particular the 1994 postscript to *The Concept of Law* ). The distinction between description and explanation also surfaces in some places. Thus, Gilbert (2011) anchors her study of promises on two “intuitive points”. The first is that “if someone has promised to do something, then he is obligated to do it, by virtue of his promise” (Gilbert, 2011). The second is that promisory obligations are *directed* at the promisee. Pinpointing these two points belongs to the descriptive social ontology of promises. In addition to these descriptive observations, Gilbert advances two further claims. First, she maintains that these intuitive points are veridical, or at least that they have some presumption of truth: she thus endorses a foundationalist ontology of promises. Secondly, she proposes a reductive analysis of promises in terms of joint commitment and argues that this analysis vindicates these intuitive observations. One virtue of making these descriptive claims explicit and distinguishing them from foundational claims, is that disagreements concerning the description of the phenomena that are to be explained can then be easily marked off from disagreements concerning the proper explanation of these phenomena. Haslanger (1995) opposes manifest concepts to operative ones. The manifest concept of coolness, for example, is that of an intrinsic property of some individuals; the operative concept of coolness is that of an extrinsic property of individuals determined by the evaluation of an in-group. The operative concept “debunks” the manifest concept, showing it to be an illusion. Haslanger’s description of the manifest concept of coolness belongs to descriptive social ontology; Haslanger’s account of the operative concept of coolness belongs to explanatory, or more precisely to revisionary, social ontology. The descriptive/explanatory distinction is also at play in Horden & López de Sa (2021)’s recent analysis of groups. Identifying groups with pluralities, Horden and López de Sa face the objection that different groups may turn out to be co-extensive. They grant that there is an “appearance of distinct but coextensive groups” (Horden & López de Sa, 2021), a claim that belongs to descriptive social ontology, but then proceed to put forward two explanatory claims. First, they argue that the appearance in question is deceptive: for it would lead us to say, for example, that, if Paul is Dean of the Faculty and the husband of Mary, the husband of Mary is not the Dean of the Faculty. Once the deceptive nature of the appearance has been diagnosed, an explanation for why it occurs can then be given: namely, in a nutshell, by recognizing that pragmatic appropriateness has been conflated with truth. These latter two claims belong to revisionary social ontology. In all three cases—Gilbert’s foundationalism about promises, Haslanger’s distinction between manifest and operative concepts; and Horden and López de Sa’s revisionism about co-extensive groups—explanatory social ontology is dependent on a prior description of social appearances.

### Social appearances

While the appeal to appearances may not strike us as especially puzzling in the philosophy of mind, where consciousness or internal perception may be thought to provide us with some access to mental phenomena, the assumption that social phenomena appear to us in a particular way may strike us as less natural. In our view, however, such a skeptical attitude arises from an overly narrow understanding of social appearances. Social appearances, as we conceive of them, are not tied to any specific mental faculty or mode of access. There are clearly many claims about the social world on which we can agree prior to committing ourselves to a particular explanatory framework. The contents of these claims are social appearances. We thus think of social appearances in a very encompassing way and take some among them to be accessible to us prior to conducting any empirical studies. Here are a few examples of candidate descriptive social claims: *it appears to be the case that…*

1. A city is not a duck.
2. Inflation cannot laugh.
3. No discussion is a president.
4. It is possible to have money in one’s pocket.
5. Ownership is a two-place relation between a person and a thing.
6. Promises generate claims and obligations.
7. A claim ceases to exist when its content is realized.
8. One can renounce a claim but one cannot renounce an obligation.
9. Revolutions, tournaments, and economic crises are occurrents.
10. Theaters, countries, and civil codes are continuants.
11. Property rights can be transferred.
12. Money can be transferred.
13. Goods can be owned.
14. Services cannot be owned.
15. Money can be owned.
16. Teams, families, and committees have members.
17. Money, constitutions, and wars have no members.
18. Some social phenomena which lack members have participants, e.g., wars, meetings, and tennis games.
19. Some social phenomena have neither members nor participants, e.g., money and civil codes.
20. Some social phenomena are fully intended (e.g., a new law, a building); some are partly intended (e.g., states, cities); and some are fully unintended (e.g., traffic jams, pollution).
21. Families, masses, communities, and societies are distinct kinds of groups.

Some of these descriptive claims, it should be noted, contain theoretical terms (e.g., “two-place relation”, “occurrents”, and “continuants”). As we will explain further in Sect. 5.1 and 5.3, we do not consider the occurrence of theoretical terms in a descriptive claim to be a problem for our proposed distinction between the descriptive and the explanatory: although appearances are in principle accessible to the keen observer, a thorough description of them may often require appeal to theoretical terms. We will take up the concern that such descriptive claims may strike us as purely philosophical or trivial in Sect. 5.3.

Some other descriptive claims require controlled studies or complex observations. Thus, data gathered by social scientists (e.g., concerning such phenomena as extreme global poverty, racial prejudice, the stock market, the under-representation of certain groups, or the occurrences of historical events) can supply descriptive claims that stand in need of an explanation. Such descriptive claims sometimes clash with common beliefs, which themselves deliver descriptive claims. To illustrate, consider the claim that, in the last 20 years, the share of the global population living in extreme poverty has increased. 52% of respondents surveyed across the world accept this claim (Roser & Nagdy, 2014; Ipsos, 2017). This result clashes with the available data which shows that extreme poverty has in fact decreased during the period in question. When there is a conflict between two descriptive claims, one needs to leave the descriptive inquiry and move to an explanatory investigation in order to settle the dispute in question. In the specific case just cited, the obvious move is (i) to revise the common belief that extreme poverty has increased and to explain why most people falsely believe this to be the case; as well as (ii) to endorse the informed descriptive claim that extreme poverty has dropped and to try to uncover the factors that led to this decrease. Thus, tensions between folk theories and empirical data (e.g., inaccurate stereotypes or misperceptions) as well as, more generally, tensions between conflicting descriptive claims can arise at the level of descriptive social inquiry and call for a resolution within explanatory social inquiry.

### Descriptive social ontology vs. descriptive social science

The distinction we have just introduced between description and explanation cuts across all domains of social inquiry, and applies not only to social ontology, but also to the social sciences. This raises the following question: what distinguishes descriptive claims in social ontology from descriptive claims in the social sciences? We regard claims (1–21) above as belonging to descriptive social ontology. By contrast, claims to the effect that inflation has increased, that racism has decreased, that extreme poverty has fallen, and statements of this sort, are all descriptive claims that belong to the descriptive part of the social sciences.

For those theorists who, like Quine (1969), take philosophy to be continuous with science, the difference between descriptive social ontology and descriptive social science will be a matter of degree. By contrast, those who want to draw a sharp distinction between descriptive ontology and descriptive empirical science can endorse two main strategies. The first is to distinguish different modes of knowledge, by arguing, typically, that descriptive claims in the social sciences require refined methods of collecting empirical data, while descriptive claims in social ontology proceed in a more a priori fashion. One problem for a proposal of this kind is that some descriptive claims advanced in the social sciences also have an a priori flavor. Thus, Menger (1892), in describing the explanandum of his study on money, remarks as follows: “We have to explain why it is that the economic man is ready to accept a certain kind of commodity [money], even if he does not need it” (Menger, 1892, p. 239). Menger did not have to establish by empirical means that economic agents exchange goods and services for money: this is an observation to which he could help himself from the start. Yet this claim, one may think, does not belong to descriptive social ontology. A more promising way of drawing the distinction between descriptive social ontology and descriptive social science, we submit, is to focus not on the methods by which claims are established but on the subject-matter under investigation. Descriptive ontological claims tend to target specific aspects of social phenomena, namely those that are associated with what are sometimes called *“formal”* concepts (Mulligan, 2014), which include for example such notions as essence, existence, identity, parthood, necessity, possibility, ontological dependence, ontological categories (e.g., the category of particulars, properties, continuants, states of affairs, or facts), as well as grounding or anchoring relations. (See Epstein, 2015, Chap. 5, for the distinction between grounding and anchoring.) On this proposal, the descriptive claim, “Teams appear to have members”, is a statement that belongs to descriptive social ontology only if it is construed, for instance, as making a claim about one of the formal notions just mentioned, as in, for example, “It appears to be part of the essence of teams to have members”. Although we suspect that most, if not all, the claims cited in (1–21) are tacitly essentialist descriptive claims, we will not defend this commitment in the present context (though see Koslicki and Massin, 2023, for a more thorough discussion of how a descriptive philosophical inquiry should be understood as targeting essences). Note that, insofar as essences figure within the scope of the appearance operator, such claims do not commit us to the reality of essences, only to what we might call “phenomenal essentialism”, the view that social entities appear to have essential and accidental properties. Anti-essentialists can very well accept that there are appearances about essence; these theorists will then simply adopt a revisionary stance and take such appearances about social essences to be deceptive.

In a similar vein, a thorough description of the phenomena at issue may alert us to the apparent presence of an asymmetric explanatory connection, e.g., as denoted by some occurrences of “in virtue of”, “depends”, “because”, and other such connectives. Whether the phenomena in question really are connected in this way, or what the nature of the relation in question might be, however, cannot be settled by purely descriptive means but requires an explanatory investigation. To illustrate, the descriptive claim that, as it appears, promises generate claims and obligations, cited above in (6), seems to suggest an asymmetric explanatory connection between an agent who has made a promise and the commitments the promisor has thereby incurred towards the promisee. We may use the verb, “to generate”, to mark the productive relationship which appears to obtain among the phenomena in question; but the description, as such, should be read as leaving open whether, in reality, promises, claims, and obligations are in fact related in this way and, if so, whether the relation at issue might be ontological dependence, metaphysical causation, grounding, anchoring, or some other connection.

This leads us to a central bone of contention within descriptive social ontology, and within descriptive metaphysics more broadly. Some philosophers take social appearances to have rather poor and simple contents. Thus, in the same way as Hume conceived of perceptual appearances in terms of spatio-temporal associations between simple and separable sensations, some descriptive social ontologists will limit descriptive social claims to mere correlations between what they take to be independent social phenomena. By contrast, other philosophers instead consider appearances to have a rich and complex content. Thus Stumpf (1873) and Husserl (1900), maintained, contra Hume, that in order to describe perceptual objects and contents properly, one needs to recognize not just separable parts, but also dependent parts such as colors, extensions, motions, Gestalten, and intensities. (Dependent parts, which Husserl calls “moments”, approximately correspond to what contemporary theorists call “tropes”.) Such moments are, on their view, essentially dependent. As Stumpf (1873, p. 113) puts it: “Their nature forbids them to have an isolated and independent existence from other contents in representation”.^5^ Now, in the same way as Stumpf and Husserl criticized empiricists for having neglected the complexity of visual appearances, descriptive social ontologists, such as Reinach (1989a [1913]), Scheler (1916) and Stein (1925), consider the content of social appearances to go beyond mere correlations. They maintain that the social world, as it appears to us, calls for descriptions in terms of such formal notions as essence and ontological dependence. For instance, promises, on Reinach’s account, do not just appear to be correlated with intentions and obligations: rather, Reinach takes it to be part of the essence of promises that they presuppose intentions and generate obligations.

The Humean view of the content of social appearances may explain the lack of uptake descriptive social ontology has experienced within analytic philosophy. For if all social appearances only reveal to us correlations between seemingly independent social phenomena, descriptive social ontology simply boils down to descriptive social science. On the other hand, if essence, necessity, ontological dependence, and other such formal notions figure in a proper description of the content of social appearances, then the task of providing such descriptions is not exhausted by the empirical study of social correlations. We take this disagreement between an austere and a rich conception of social appearances to constitute a further reason to undertake a careful descriptive investigation before proceeding to explanatory social ontology. This debate, in particular, should not be settled by explanatory commitments one brings to the scene prior to having engaged in a thorough description of the appearances. To deny that social phenomena appear to enter into essential connections, for example, because one fails to accept the existence of such connections, or to maintain that social phenomena present us with essential connections because one accepts their existence, would exemplify the explanatory fallacy and violate the thesis that descriptive social ontology ought to be prior to explanatory social ontology in the order of inquiry, to which we now turn.

### The priority of descriptive social ontology

Our central claim is that descriptive social ontology is not only distinct from, but also prior in the order of inquiry to, explanatory social ontology. While few philosophers in the analytic tradition explicitly embark on a thorough description of the phenomena prior to attempting to explain them, we believe that the priority of descriptive social ontology to explanatory social ontology is nevertheless largely implicitly acknowledged. A case in point is Searle’s conception of the construction of social reality, as defended for example in Searle (1995, 2010). Searle is engaged in a foundationalist project. While he makes clear from the very start that his theory must meet certain explanatory desiderata, in particular that institutional facts must be explained in a unified manner that is compatible with Searle’s naturalism, no thorough description is given by Searle of what he takes the explananda of his foundationalist theory to be, apart from a list of examples. Though Searle is not a descriptive social ontologist, his implicit acceptance of the priority of the descriptive over the explanatory nevertheless becomes apparent in how he handles objections. A number of objections have been leveled against Searle’s account. Some of these objections do not target Searle’s descriptive claims, but instead take issue with Searle’s proposed explanations of the appearances in question. An objection of this sort is leveled against Searle’s account, for example, by Hindriks & Guala (2015), who argue that constitutive rules can be reduced to regulative rules. Likewise, Tieffenbach (2010) argues on the basis of Menger (1892) that, contrary to Searle’s explanatory account, the origin of money can be explained without resorting to collective intentionality. In both cases, it is the alleged lack of parsimony of Searle’s explanatory account that is targeted by the objections in question.

Other objections, however, are not addressed to Searle’s explanatory framework, but rather highlight certain descriptive deficiencies of Searle’s account. In this vein, such phenomena as nations or electronic money (Ruben, 1997; Thomasson, 2003; Smith, 2003), whose creation does not seem to require the collective acceptance of status-function ascriptions to a physical phenomenon, have been used to make trouble for Searle’s approach. Likewise, it has been argued that unintended social phenomena or by-products of intended phenomena, such as recessions or racism (Thomasson, 2003) or business cycles (Friedman, 2006), are incompatible with Searle’s intentionalist framework. Such objections adduce *counterexamples* to Searle’s theory. Counterexamples, as we argued above, are descriptive claims, in the sense that they point to the presence of certain appearances which the theory in question fails to address adequately. To illustrate, as it appears, inflation is a social phenomenon which does not consist in the collective assignment of status-functions through declarations. If such an appearance is regarded as veridical, we arrive at a counterexample to the view that all institutional phenomena consist in the collective assignment of status-functions.

Searle (2006a, b, 2010), p. 22)’s answer to these objections consists in conceding that these considerations do indeed present counterexamples to his previous account and that, in response, his theory must be restricted in certain ways. Thus, in order to make room for unintended social phenomena, Searle introduces a distinction between “ground floor institutional facts”, such as monetary exchanges, and “systematic fallouts”, such as inflation, and maintains that his theory of institutional facts concerns only ground floor institutional facts. As long as other institutional phenomena can be viewed as “macro consequences” of these, such a restriction of his original theory, so Searle maintains, is harmless (see also Burman, 2015). This answer is unsurprising in light of our proposed understanding of the descriptive/explanatory distinction. In its reformulated form, Searle’s theory does achieve better descriptive adequacy; but it does so at a loss of explanatory power, since two explanatory posits are now needed to explain institutional facts: the imposition of status functions plus an explanatory connection between micro- and macro-phenomena. Had Searle started from a description of the distinction between intended and unintended institutional social facts or their by-products, instead of adjusting his theory afterwards to address counterexamples involving apparently unintended institutional phenomena, he might have arrived at the same theory by way of a different procedure. As long as counterexamples are treated as they are by Searle, no explanatory fallacy results, and the priority of the descriptive is maintained. The only difference is that, when a theory is adjusted retro-actively in the face of counterexamples, the description of the appearances is no longer temporally prior to their explanation. In other ways, however, the description of the appearances maintains its priority over their explanation in the order of inquiry, as long as counterexamples are not simply dismissed on the grounds that they are incompatible with the explanatory framework in question.

Searle’s revised account, however, is open to further counterexamples in cases in which systematic fallouts do not arise from institutional facts but from individual actions, independently of any collective intentionality. Spontaneously formed pathways, pollution and traffic jams constitute natural examples of the phenomenon in question.^6^ Various moves are open to Searle in response to such counterexamples. He might further restrict his theory of institutional facts; or he might reject such counterexamples by arguing that, contrary to the appearances, these cases do in fact involve some concealed collective intentionality. One move, however, that is not open to Searle is simply to dismiss such counterexamples on the basis that they do not fit his proposed explanatory framework. To argue, for instance, that given the success of the status-function account of institutional facts, traffic jam must be redescribed in such a way that they involve collective intentionality will plainly be an instance of the explanatory fallacy, i.e., of letting one’s explanatory agenda contaminate one’s descriptions.

We have argued that descriptive social ontology is prior to explanatory social ontology in the order of inquiry. But what about practical social ontology? Should critical or ameliorative considerations not take precedence over descriptive ones? We think not, although we cannot argue for this stronger claim in the present context. In our view, theoretical social ontology—descriptive social ontology together with explanatory social ontology—should similarly take precedence in the order of inquiry over practical social ontology in the following sense. In order to determine whether a certain condition or feature of the social world needs to be improved, whether it should be improved, or in which direction and how it can be improved, one must first arrive at a clear vision of what the condition or feature in question is and what moral or political value(s) it actually has. Any attempt to improve or preserve a certain condition or feature relies on hypotheses, be they tacit or explicit, about its nature and worth. Even the project of “de novo conceptual engineering”, recently proposed by Chalmers (2020), which advocates building new concepts in place of fixing old ones, presupposes a careful description and evaluation of the concepts that are already in place. One reason for this is that the very claim that a concept is new presupposes that it is not already included among the familiar concepts that are in our possession to begin with. This latter assessment requires that the concepts that are already in place be carefully studied descriptively before they are replaced by new ones.

## Objections

In what follows, in order to clarify further the distinction we have proposed to draw between descriptive and explanatory approaches, we consider the following four objections. First, given the theory-ladenness of observation, one might wonder whether a distinction between description and explanation can really be drawn if any attempt at describing the appearances already implicates theoretical commitments. Secondly, although descriptions can apparently sometimes be revised on the basis of insights gained through explanation, it is not immediately obvious how or whether our approach makes room for this possibility. Thirdly, if appearances are immediately accessible to any inquirer who cares to look, we might expect that descriptive investigations are likely to yield only trivial or uninteresting results. Fourthly, on sensitive issues such as gender and race, political biases appear to be insurmountable, so that the idea of sharply distinguishing theoretical from practical social ontology might seem to be doomed to fail.

### The theory-ladenness of observation

The distinction between the descriptive and the explanatory should not be conflated with the distinction between what is “pre-theoretical” or “theory-neutral”, on the one hand, and what is “theoretical”, on the other hand. While there may be some descriptive statements (e.g., “I am older today than I was yesterday”) which strike us as plausible independently of any particular theory within which they are embedded, it should not be assumed in general that descriptive statements can always be formulated in an entirely theory-neutral way.

The first reason why descriptive metaphysics need not be committed to theory-neutrality is that, although the contents of appearances may be theory-neutral, the descriptions of these contents may require the introduction of new concepts and terms which are not already available in our everyday conceptual scheme or ordinary language prior to the attempted description. As Cappelen (2020, p. 147) puts it, “a ‘pure’ descriptivist would show a lack of understanding for the need to assess and improve on the concepts used to engage in the descriptive project”. We fully agree, and so do many descriptivists in effect. To illustrate, the following distinctions and concepts have been introduced in Brentano’s school, among many others, to describe appearances properly: the distinctions between presentations and judgments (Brentano, 1874); between affective sensations such as pains, itches or bodily pleasures, and emotions such as suffering, annoyance or enjoyment (Stumpf, 1928); between simple and complex colors (Brentano, 2009, pp. 127–160), between actions and their products (Twardowski, 1912), or between values and norms (Meinong, 1917); the concepts of representational content (Twardowski, 1977); of ontological dependence (Stumpf, 1873; Husserl, 1900); of Gestalt (Ehrenfels, 1890); or of boundaries (Brentano, 1988). None of these distinctions and concepts are uncontroversial; but each of them was introduced for a descriptive purpose; and some of them, such as the concept of ontological dependence, have been widely re-used in explanatory metaphysics following their introduction for descriptive purposes.

As a further illustration of the distinction we have in mind, consider for example the phenomenon of akrasia. Suppose we characterize an akratic agent, as neutrally as possible, as an agent who, in some sense, acts against their own better judgement. Even while still being engaged in the descriptive project of merely stating what the phenomenon of akrasia seems to consist in, we may find it necessary to take recourse to such terms as “action”, “intention”, “motivation”, “evaluation”, and “judgment”, which may eventually go on to play a central role in an explanatory account of what goes on in akratic agents. Nevertheless, we should distinguish a mere description of what the phenomenon of akrasia seems to consist in from an explanation of what in fact goes on within an akratic agent, even when the descriptive statement already makes use of vocabulary which comes to be assigned a theoretical role within a particular proposed explanatory account. The descriptive phase targets the sense in which, for example, the Socratic, Platonic, Aristotelian, and Davidsonian account of akrasia all aim at providing a solution to a single problem, regardless of which precise terms are chosen to state the problem in question.

Secondly, descriptive metaphysics may be theory-laden in a stronger sense: the contents of the appearances themselves may be shaped by concepts. This broadly Kantian insight can take various forms (e.g., the critique of the myth the given; the Sapir-Whorf hypothesis; as well as the positing of conceptual content; cognitive penetration; the theory-ladenness of observation; etc.), and controversies surrounding such issues persist. Descriptive metaphysics is compatible with such top-down influences. Thus, Strawson mentioned Kant, along with Aristotle (in opposition to Descartes, Leibniz and Berkeley), as prominent descriptive philosophers (Strawson, 1959, p. 9). One should here distinguish the view that the appearances are shaped by shared concepts, such as social categories, from the view that the appearances are shaped by the very theoretical conceptualizations that are introduced by the descriptive metaphysician. The former view raises no special worry. What carves the appearances, in Kant’s view, are the a priori forms of sensibility and the a priori categories of the understanding, not, obviously, his critique of pure reason. This latter view, however, would pose a more serious challenge for those who want to maintain a distinction between the descriptive and the explanatory: if the descriptive metaphysician’s concepts shape the contents of the appearances he sets out to describe, it could be argued that descriptive metaphysics is not so much in the business of describing appearances that are already there, but rather creates the appearances it claims to describe. Such a strong kind of theory-ladenness would indeed be incompatible with the project of descriptive metaphysics, but it would also be intrinsically problematic for the very reason adduced in favor of descriptive metaphysics. Indeed, an explanation that generates its own explanandum seems to lack an independent criterion of evaluation and, consequently, threatens to be trivially successful.

### Immunity to revision

Secondly, our account might appear to rule out situations in which a description of the appearances would need to be revised on the basis of insights gained through explanation. For in enforcing a distinction between what is descriptive, on the one hand, and what is explanatory, on the other hand, and in barring any influence of explanation on description, we might seem to be committed to rendering the descriptive immune from revision on the basis of explanatory insights. We have earlier granted the possibility of revising descriptive claims, for example, in cases in which we are presented with a seeming conflict between appearances (e.g., as when extreme global poverty is both thought to have increased and decreased). It is worth considering, however, whether descriptive claims might not also be subject to revision for other reasons.

Consider, for instance, the claim: “As it appears, all instances of jade are of the same kind”. On the face of it, this statement strikes us as a purely descriptive claim concerning instances of what is ordinarily referred to as “jade”. At the purely descriptive level, instances of what we commonly refer to as “jade” share certain similarities with respect to their phenomenal qualities, including for example their predominantly green color. These apparent similarities at the phenomenal level might tempt us to conclude that there is a single kind of mineral, *jade*, to which all jade-instances belong. When we attempt to explain why jade-instances are similar to one another in these respects, however, it turns out that their superficial resemblance can in fact be traced to two quite different underlying micro-structures which come to be associated with two distinct kinds of minerals, *jadeite* and *nephrite*. Does this case present us with a situation in which the descriptive statement with which we began is subject to revision on the basis of insights that are gained only once an explanatory framework is in place?

Let us suppose that we initially accept the appearance that all jade-instances belong to the same kind as veridical, and set out to explain why all instances of what we ordinarily call “jade” are similar to one another with respect to their phenomenal qualities. In the course of looking for an explanation, we discover that, contrary to our initial inclination, it is not in fact the case that there is a single kind of mineral, *jade*, to which all instances of what we ordinarily call “jade” belong. This new piece of evidence does not in itself provide us with an explanation of the initial appearance: facts that are discovered in the course of looking for an explanation of a phenomenon do not necessarily themselves provide an explanation of the phenomenon in question. Rather, the discovery that all instances of what we ordinarily call “jade” do not belong to a single kind of mineral actually contradicts the initial appearance that led us to believe that they do.

When we encounter a situation of this type, we might attribute the mismatch in question not to the *appearance* itself, but rather to the initial *description* of the appearance: if this description is read as committing us to the existence of a single kind of mineral, *jade*, to which all instances of what we ordinarily call “jade” are supposed to belong, then we might regard the initial description of the appearance as defective in illicitly going beyond what is strictly speaking presented to us by the appearance itself. Given this option, the appearance in question, once accurately described, only licenses the descriptive claim that all instances of what we ordinarily call “jade” share certain phenomenal qualities, including their predominantly green color, without taking the further step of positing a single homogeneous kind of mineral, *jade*, whose alleged unity is somehow thought to underwrite the superficial similarities that appear to us. Thus, given this reaction, a faulty descriptive claim to the effect that instances of what we ordinarily call “jade” belong to a single kind of mineral, *jade*, should be rejected in favor of a more accurate descriptive claim to the effect that instances of what we ordinarily call “jade” are similar in certain respects (e.g., their predominantly green color).

This response illustrates that our approach can make sense of the idea that one’s attitude towards one’s initial description of an appearance may need to be adjusted in light of insights that are gained only through explanatory advances. At no point in this process should we ever expect to find ourselves in a situation in which an explanans conflicts with its explanandum: for we take it to be an unassailable principle governing explanation that if a phenomenon A explains a phenomenon B, then A and B cannot contradict each other. Nevertheless, in the course of searching for an explanation, we may very well discover that it is necessary to re-appraise what we initially took the explanandum to be.

### Triviality

If descriptive claims simply aim to capture the world as it appears to us, one might be inclined to think that such an inquiry could only ever lead to results that are trivial or uninteresting. For assuming that the appearances are immediately accessible to anyone who cares to look, then why, so one might wonder, is it necessary for us to embark on a lengthy descriptive investigation only then to end up with insights that were already within our reach from the very beginning? In response to this concern, we want to highlight that, despite the fact that the appearances are in principle accessible to us, a descriptive project can nevertheless lead to significant advantages arising from, first, the inherent *difficulty* of attaining an accurate and complete description of the appearances; secondly, the *importance* of correctly identifying the phenomena that an explanatory framework needs to address; and, thirdly, the *usefulness* of the descriptive task in potentially spotting deficiencies or tensions in a proposed explanation of the phenomena in question.

First, while we agree that the appearances are (or at least should be) accessible to a keen observer from the start, it can nevertheless be a remarkable achievement to arrive at an accurate and complete description of the appearances: for what is familiar to us can sometimes be difficult to notice, precisely because we are so accustomed to it. We find Reinach voicing a thought along these lines in the following passage:

One could no longer doubt the existence of an a priori sphere if one clearly realized what a vast multitude there is of such self-evident legal rules, which, although they are nowhere formulated, are naturally and easily applied, and which we usually do not become fully aware of only because they make so much sense and are so immediately understandable. (Reinach, 1989a, 273; English translation, p. 135).

Secondly, even if a descriptive approach merely makes explicit what should have already been obvious to a keen observer from the start, the significance of this activity can nevertheless at times be considerable. To illustrate, claims such as “A city is not a duck” as well as others cited earlier in (1–21), may initially strike us as, in a sense, so obvious that it is not worth making them explicit. However, once we identify an apparent triviality that is tacitly presupposed by everyone concerned with the phenomena in question, this observation can then in turn be used as a data point against which to measure the success of a proposed explanatory framework that is directed at the phenomena in question. Thus, Massin and Tieffenbach (2017) argue on just such grounds that an account of economic exchanges which contradicts the seemingly trivial observations such as those stated in (14) and (15), that money can be owned but services cannot be owned, should be rejected. Despite the intuitive plausibility of this desideratum, as Massin and Tieffenbach note, an explanatory account of economic exchanges which validates this descriptive starting-point is surprisingly hard to come by.

Thirdly, the benefits of arriving at a complete and accurate description of the phenomena in question, even if such an endeavor sometimes merely consists in making explicit seemingly obvious tacit presuppositions, becomes apparent when we consider the usefulness of descriptive claims in highlighting potential ambiguities or other problems that may affect a proposed explanatory framework. Outside of social ontology, the potential usefulness of descriptive claims for steering one’s explanatory outlook in the right direction can be illustrated, for example, by reference to Fine’s celebrated point that essence is not reducible to necessity (Fine, 1994). This claim is, at least in part, descriptive in nature and is supported by Fine through the use of various influential counterexamples which appear to make trouble for modal conceptions of essence. Counterexamples, as we argued above, should be understood as descriptive, rather than explanatory, claims. Thus, when we consider the world as it presents itself to us in naïve terms, the fact that Socrates is necessarily the sole member of Socrates’ singleton set does not strike anyone, who is not already in the grip of a particular explanatory framework, as having any *prima facie* relevance to Socrates’ identity as a person. This much is part of simply describing how the world appears to us in naïve terms. Once it has been appreciated, however, that what is necessarily the case can apparently diverge in this way from facts concerning the essence or nature of a thing, this intuitive realization can then pave the way for an explanatory framework which resists the temptation to reduce essence to necessity. The foundational move towards a non-modal conception of essence, therefore, has its origin in a descriptive insight, which belongs to the domain of naïve metaphysics, that essence and necessity appear to part ways in the manner highlighted by Fine.

Within the domain of social ontology, the potential usefulness of descriptive claims to clear up explanatory entanglements can be illustrated by means of the account of money offered in Guala (2021). Guala proposes an explanatory framework according to which money should be seen primarily as a type of institution and only derivatively as a type of object. Following the more general account of institutions developed in Hindriks & Guala (2015), Guala takes the institution of money to be a system of rules-in-equilibrium which regulates how people engage in economic transactions. This explanatory account runs into several potential conflicts with prima facie plausible descriptive claims, which seem to privilege the conception of money-as-an-object over Guala’s preferred conception of money-as-an-institution. To illustrate, according to (4), (12), and (15), cited earlier, we can carry money in our pockets as well as own and exchange it. Evidently, however, we cannot carry systems of rules in our pockets; nor are systems of rules themselves what we own or exchange in economic transactions. When faced with these apparent conflicts, Guala has several options. First, he might choose to endorse a revisionary approach, as he seems tempted to do, and claim that the apparent priority of money-as-an-object over money as-an-institution is illusory. A second strategy that is open to Guala, however, is to refine his descriptive ontology of money and shift the target of his explanatory account to what we might call the “monetary system”, i.e., the system of rules which *institute* money. Given this second response, Guala can now interpret the above-mentioned descriptive claims as concerning money in the sense of what *is instituted by* the monetary system. Moreover, it is this latter conception of money as what *is instituted by* the monetary system which seems more closely aligned with the functional roles which, as Guala acknowledges, are typically ascribed to money by economists, viz., that money serves as a medium of exchange, store of value and unit of accounting (Guala, 2021, p. 3).^7^ This potential shift in the primary focus of Guala’s explanatory framework further supports our central claim that a thorough, accurate, and unbiased description can lead to clarifications and improvements in an explanatory account of social appearances.^8^

### Political biases

Above, we addressed the objection that descriptions should be theory-neutral, by arguing that introducing theoretical concepts and distinctions for the purposes of capturing the appearances accurately is fully compatible with, and indeed congenial to, the descriptive project. The following closely related, but distinct, objection remains yet to be addressed: Is a politically neutral description of social appearances even possible?^9^ If not, then it may appear to be impossible to pursue the theoretical project of attempting to describe or explain social appearances without also simultaneously engaging in the practical project of striving to preserve or improve features of the social world. For if the description of social phenomena can never be insulated from the inquirer’s political commitments, then the independence we have claimed for theoretical social ontology vis-à-vis practical social ontology also might seem to be jeopardized. In the preceding remarks, we have deliberately focused on such examples as money and contractual obligations, while avoiding such cases as gender and race, which appear to be more explicitly politically loaded. This, however, should in no way be taken to imply that these latter phenomena are any less worthy of being theoretically investigated. Our intention, in proceeding in this way, was simply to facilitate the task of distinguishing the theoretical from the practical project.^10^

But how, then, would the descriptive social ontologist approach other more sensitive cases, where the theoretical inquiry seems to be inextricably linked to one’s practical goals? There is an important truth in this objection: political biases are indeed ubiquitous and particularly strong when it comes to sensitive issues such as those surrounding race or gender. But the objection ultimately relies on a non-sequitur. From the strength and ubiquity of political biases we may only infer that a politically un-biased descriptive social ontology is difficult to carry out, not that its achievement is impossible or undesirable. The widespread sentiment that “everything is political”, if accurate, does not entail that scientists and philosophers should stop striving to curb their political prejudices when engaged in theoretical projects. Quite the contrary: the fact that confirmation biases are so commonly found in contexts in which questions of political relevance are debated, if anything, provides an even stronger reason to try to neutralize their interference. Although no doubt difficult, progress in this direction can be made in a variety of ways, including the following. First, while individual attempts at overcoming one’s own political biases often fail, collective endeavours to increase neutrality by cultivating viewpoint diversity and disagreement have been shown to be both effective and achievable (Wang et al., 2019; van Veen et al., 2020). Second, keeping in mind the distinction between theoretical and practical social ontology may contribute to our ability to pursue social ontology without becoming enmeshed in a political battlefield. If the domain of theoretical inquiry is recognized to be both important on its own terms and distinct from the pursuit of practical goals, the temptation to let our political inclinations influence our descriptive and explanatory endeavours can be at least to some extent resisted.

## Conclusion

In this paper, we have put forward a plea for a descriptive approach to social ontology, which has often taken a back-seat to the development of explanatory frameworks. If our account is on the right track, explanatory commitments should be at least initially bracketed if we are to arrive at an adequate non-biased description of social phenomena. To help motivate the proposed distinction between description and explanation we considered, first, an analogy from the philosophy of mind where we similarly detect two different ways of investigating mental phenomena: a descriptive approach taken by descriptive psychology (otherwise known as “phenomenology”) and an explanatory approach utilized in analytic philosophy of mind which focuses primarily on the resolution of the mind/body problem. Next, we situated our proposed distinction between description and explanation in relation to other similar positions in general metaphysics as well as philosophy at large, such as those proposed, among others, by Aristotle, ordinary language philosophers following Wittgenstein, Strawson, and, more recently, Fine.

We advanced two central claims in this paper: first, that description and explanation provide us with two distinct ways of engaging with social phenomena; and, secondly, that the descriptive project ought to be prior to the explanatory project in the order of inquiry. An accurate unbiased description of the phenomena, we argued, is needed to provide an explanation not only with its target explanandum, but also with the criteria of success by which an explanation, once it has been formulated, can be evaluated. Among explanatory approaches, we distinguished between foundationalism, on the one hand, and revisionism, on the other hand: the former approach takes the appearances to be veridical and then sets out to explain their content; the latter approach takes appearances to be deceptive in some way and must then explain why these illusory appearances present themselves to us.

Disputes can arise not only between the different explanatory postures, but also from within the descriptive camp. We identified two important kinds of descriptive disagreements. The first such disagreement concerns the relative degree of richness ascribed to the content of appearances: those, on the one hand, who take a broadly Humean approach consider the content of appearances to be rather minimal, consisting typically of associations of independent elements; those, on the other hand, who take a broadly Brentanian approach ascribe to the appearances a much richer content, which might for example include relations of essence, parthood, or ontological dependence. The second kind of descriptive disagreement concerns the orthodoxy or heterodoxy of the descriptions that are given. In our view, heterodox descriptions, such as Berkeley’s claim concerning the non-existence of matter, for example, should not be conflated with revisionary explanatory claims, since a disagreement about the right description of an appearance must be kept distinct from a disagreement about the veridicality of an appearance.

With these clarifications in hand, we proceeded to apply our proposed distinction between description and explanation to the social domain. In this realm as well, we argued, we have available to us a wide array of descriptive claims concerning social phenomena, such as money, cities, groups, laws, promises, obligations, and the like, which often strike us as prima facie plausible, independently of any commitments to a particular explanatory framework. Although some of these claims might initially seem to us so trivial that they are not even worth stating explicitly (e.g., “We can carry money in our pockets”), such tacit presuppositions can nevertheless supply valuable data points against which to evaluate the success of an explanatory framework.

An important pending issue concerns the question of whether social appearances should be construed as having a minimal or a rich content. One reason, we suspect, why descriptive social ontology has been rather sidelined within the analytic tradition is the tacit assumption that descriptive social claims only report empirical data and statistical correlations. We are doubtful that this thin construal in fact yields the correct conception of the content of social appearances. Rather, it strikes us as plausible to think that a proper description of the contents of social appearances will have to avail itself of a philosophically substantive apparatus, including such formal notions as essence, ontological dependence, and parthood. Just as an appeal to such formal concepts is needed to arrive at a proper description of how, for example, color, extension, location and objecthood are related to one another in visual experience, so we believe that the social world as it appears to us is similarly imbued with a rich array of connections and distinctions whose proper description will require an elaborate descriptive social ontology. A proper defense of this commitment to a rich conception of the contents of social appearances, however, will have to await a separate treatment in its own right (see Koslicki and Massin, 2023).

Our primary aim in this paper has been to argue that descriptive social ontology ought to be regarded as prior to explanatory social ontology in the order of inquiry, i.e., that we should aim to describe how the social world appears to us prior to trying to explain it. The distinction between descriptive and explanatory social ontology, we proposed, belongs to the domain of theoretical inquiry. The latter domain, in turn, should be distinguished from practical social ontology which aims at preserving or changing the social world, e.g., by repairing or improving it in certain respects. It furthermore strikes us as plausible that theoretical social ontology similarly ought to be regarded as prior to practical social ontology, in the sense that trying to discover what the social world is like would seem to be necessary for any attempt at preserving or improving it. A defense of this latter claim, however, concerning the priority of the theoretical over the practical lies outside of the scope of the present discussion.^11^

## Footnotes

1 According to the Philpapers 2020 survey (Bourget & Chalmers, 2022), about 57% of philosophers who specialize in the philosophy of mind accept or lean towards naturalism, while only 24% accept or lean towards non-naturalism. Since there was no category for a specialization in phenomenology or descriptive psychology in the survey, the closest category to consider is that of “continental philosophy”: 27% of philosophers specializing in continental philosophy accept or lean towards naturalism, while 48% accept or lean towards non-naturalism.

2 We are grateful to an anonymous referee of this journal for raising this objection.

3 We are grateful to an anymous referee of this journal for alerting us to the significance of this point.

4 See Mulligan (1987) and Schumann & Smith (1990) for a discussion of the relations between the theories of social or speech acts proposed by Reid, Reinach, Austin, and Searle.

5 Note that, as attested by the phrase, “in representation”, this claim is only committed to what we have earlier called “phenomenal essentialism”: Stumpf aims primarily at describing perceptual appearances (see Smith and Mulligan, 1982, p. 39); we take it that these claims are under the scope of a tacit “as it appears” operator.

6 Searle’s revised account may even fail to apply to the phenomena it was meant to accommodate, such as business cycles or inflation. For suppose Menger (1892) is right that money can arise in the absence of any collective intention; then, inflation could arise without requiring collective acceptance, even at the ground level. Moreover, given Menger’s account, money does not bring with it any deontic powers (see, again, Tieffenbach, 2010).

7 See also Passinsky (2020) for a distinction between a descriptive and a normative approach to the question of whether bitcoin should be classified as money. Passinsky’s more general hylomorphic approach to social kinds is discussed in Passinsky (2021).

8 We would like to acknowledge the extremely helpful feedback we received from Francesco Guala when we presented him with some of the points raised in the text (personal communications).

9 We are grateful to two anonymous referees of this journal for raising this worry.

10 Regarding the question of what should or should not count as a phenomenon that is paradigmatic or peripheral to the study of social ontology, see for example Ásta’s critique of Ian Hacking’s exclusion of gender (Ásta, 2013, 2018; Hacking, 1999).

11 We would like to thank Francesco Guala and Raphaël Künstler for helpful comments on this paper. In addition, we are grateful to feedback we received from the referees as well as audiences at Central European University, the Metametaphysics of Social Ontology and Synthese Special Issue conference, as well as the International Social Ontology Conference.
